# Supplementary figures and images for: Overcoming quadratic hardware scaling for a fully connected digital oscillatory neural network
Source: Front Neurosci. 2026 Jan 15;19:1658490. doi: 10.3389/fnins.2025.1658490 (PMC12851967; doi:10.3389/fnins.2025.1658490)

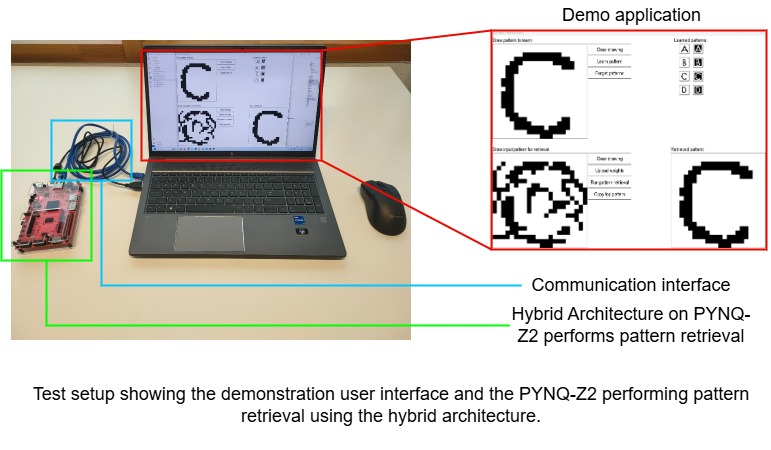

Supplement: Supplementary file 1 [file Image_1.jpeg]
